# A New Practical Formulary of Hospitals of England, Scotland, Ireland, France, Germany, &c.

**Published:** 1836-07

**Authors:** 


Art. XII.—*A new practical Formulary of Hospitals of England, Scotland, Ireland, France, Germany, &c.* Translated from the new French edition of MM. Milne Edwards and P. Vavasseur, and considerably augmented, by Michael Ryan, m.d. London, 1835. A pocket vol. of 571 pages.

Drs. Edwards and Vavasseur have given in this little work a general dispensatory, comprising most of the medicinal substances used throughout Europe, as well as many of the formulæ adopted in the principal hospitals; to these the translator has made some additions, consisting in great part of the formulæ which he employs in his own practice. The book certainly contains many drugs and many prescriptions; but the translator somewhat too heedlessly tells us, at page 3 of his preface, that "it is a modern universal pharmacopœia, and contains a vast number of new remedies, seldom if ever employed in this country, as well as many thousand medicines that have been omitted in our Pharmacopœias." And again, at p. 5, that "the names of many thousand medicines, seldom employed in this country, were given in popular French;" and that it was a troublesome undertaking to translate them. His own index shows that the sum total of drugs in the book is about 900, of which many appear there twice under different names. Yet this is a useful compendium; and its value to beginners would have been still greater had the slips of the pen or of the press been less numerous. At p. 251 we are told that sulphate of potassa is an energetic stimulant, that it is a violent poison in large doses, &c. &c.; meaning, of course, the sulphuret of potassa: and the same mistake occurs in several subsequent pages. In like manner, at p. 254 we have the sulphate of soda and sulphate of lime instead of the sulphuret of soda and sulphuret of lime.

At p. 306, and in several other places, we find bichlorate of mercury put for bichloride of mercury; and at the same page "mercury in astringent vegetables" is stated to be one of the substances incompatible with corrosive sublimate.

The following lozenges are certainly extravagant; we should not like to give them to any of our children.

"Lozenges of Cream of Tartar and Manna. H. of Germ.

℞. Potassæ supertartratis, ℥ ss;
Mannæ . . . ℥ iv.
Aquæ . . . ℥ x.

M. Boil to a proper consistence, and make into two lozenges.
Used as a laxative for children."—(P. 497.)

So also, the following absorbent powder is said to be used in the German hospitals, but, to us, it savours much more of Brobdignag than Germany.

"℞. Magnesiæ calcinatæ, ℥ ss;

Corticis aurantii,

Sacchari, āā ʒ j.

Divide in chartulas ij, capiat iij vel iv. quotidie."—(P. 500.)

At p. 422, Dr. Ryan, when describing the method of obtaining narceine, says, "this is submitted to pressure, heated with boiling alcohol, 40° Rem.;" &c. Now 40° of Reaumur = 122° of Fahrenheit, a temperature far below that at which alcohol boils. The translator has here misunderstood Magendie, who says "on le soumet à la presse, puis on le traite par l'alcohol à 40° bouillant;" &c. (Formulaire, 8vo. edit. p. 78.) The 40° indicates the specific gravity of the alcohol as measured by Baume's hydrometer, being about the strength of the sp. rectificatus of the London pharmacopœia.

It must, no doubt, seem ungrateful to an industrious translator to dwell almost exclusively on minute faults; but in a practical formulary minutiæ are of all things the most important. The use of such a work as the present will, we apprehend, be found the greatest by those whose practical knowledge assists their judgment in estimating the respective value of the numerous formulæ thus placed before them. An extract from the eighth chapter, which contains the alteratives, will show the plan of the work more clearly.

"*Antivenereal Pills.* H. of Montp.

℞. Auri et sodæ chloruret. gr. ¼;

Extract. saponariæ, ʒ i;

Pulv. gum. acaciæ, q. s.

Fiant pilulæ xxx, quarum capiat i—viij quotidie.
Each pill contains gr. 1-60th of the chloride.
Externally. Gr. 1-15th mixed with starch, in frictions in the interior of the mouth.

*Powder of the Chloride of Gold and Sodium.* H. of Montp.

℞. Chlor. auri et sodii, gr. i;
Pulv. iridis florent, gr. ij.

Misce intime, et divide in chartulas, xv.
One of these powders should be used at each friction.

*Pommade of the Chloride of Gold and Sodium.* (Magendie.)

℞. Chlor. auri et sodii, gr. x;
Adipis præpar. ℥ss.

Fiat unguentum.
*This pommade is applied to the surface of a small blister, to cause an absorption of the salt of gold.*"—(P. 359.)

"ARNICA. (FLOWERS AND ROOT.)

A very active stimulant to the nervous system. It is used in chronic rheumatism, paralysis, and amaurosis. It is also recommended as a febrifuge. The flowers pulverized act powerfully.

*Subst. Incompat.* The sulphates of iron and zinc, the acetate of lead, the mineral acids, &c.

INTERNALLY. Flowers. *Powder,* gr. vj—x. and gradually increased xxx—vj.

*Decoction and Infusion,* ʒ j—iv to Oij of water. They should be filtered through paper.

The root. *Powder,* gr. xij—ʒ i.

*Decoction.* The same as the flowers.

*Tincture étherée.* P. ℈i—ʒ ss in a potion.

*Infusion of Arnica.* Hot. D. H. de la Ch.

℞. Florum arnicæ montanæ, ʒ j;

Aquæ ferventis, Oij.

Cola per chartam.

*A teaspoonful to be taken at a time and frequently repeated. An ounce of the syrup of orange peel may be added to this infusion. It is employed in certain cases of apoplexy, paralysis, &c.*

*Nervine Infusion d'Arnica.* H. of Italy.

℞. Radicis arnicæ mont. ʒij;

Aquæ ferventis, q.s.

Coque ad ℥ vj, per chartam chola et adde,

Etheris sulphurici, ℈ ij.

*The dose is a spoonful at a time.*

*Vinous Infusion of Arnica.* H. of Germ.

℞. Flor. arnic. mont. ℥ ss;

Aquæ puræ, Vini albi, āā ℥ vj.

Coque, cola, et adde,

Syrup. cort. aurant, ℥ ss. Misce.

*The dose is half a glassful every hour.*

*Compound powder of Arnica.* H. of Germ.
℞. Pulv. rad. arnicæ, Pulv. rad. serpent., Olei menth. piper. āā ʒ ij.
Divide in chartulas vj, quarum capiat unam, secundâ quâque horâ.

*In severe fevers accompanied with diarrhœa.*

*Stimulating and Tonic Bolus.* H. de Montp.
℞. Pulv. flor. arnicæ, Camphoræ, āā gr. iv;
Theriacæ, q. s. Fiat bolus.

EXTERNALLY. *Decoction.* In lotions, fomentations, &c." (P. 370.)

Upon the whole, we can very properly recommend this work not only to our readers in general, but to those medical men in particular who amid the wear and tear of practice sink into a hurtful sterility of prescription, and take up the convenient doctrine that there are only half a dozen good medicines in the world.

